# Feature Selection and Classifier Parameters Estimation for EEG Signals Peak Detection Using Particle Swarm Optimization

**DOI:** 10.1155/2014/973063

**Published:** 2014-08-19

**Authors:** Asrul Adam, Mohd Ibrahim Shapiai, Mohd Zaidi Mohd Tumari, Mohd Saberi Mohamad, Marizan Mubin

**Affiliations:** ^1^Applied Control and Robotics (ACR) Laboratory, Department of Electrical Engineering, Faculty of Engineering, University of Malaya, 50603 Kuala Lumpur, Malaysia; ^2^Faculty of Electrical Engineering, Universiti Teknologi Malaysia, 81310 Johor Bahru, Malaysia; ^3^Faculty of Electrical and Electronic Engineering, Universiti Malaysia Pahang, 26600 Pekan, Pahang, Malaysia; ^4^Faculty of Computing, Universiti Teknologi Malaysia, 81310 Johor Bahru, Malaysia

## Abstract

Electroencephalogram (EEG) signal peak detection is widely used in clinical applications. The peak point can be detected using several approaches, including time, frequency, time-frequency, and nonlinear domains depending on various peak features from several models. However, there is no study that provides the importance of every peak feature in contributing to a good and generalized model. In this study, feature selection and classifier parameters estimation based on particle swarm optimization (PSO) are proposed as a framework for peak detection on EEG signals in time domain analysis. Two versions of PSO are used in the study: (1) standard PSO and (2) random asynchronous particle swarm optimization (RA-PSO). The proposed framework tries to find the best combination of all the available features that offers good peak detection and a high classification rate from the results in the conducted experiments. The evaluation results indicate that the accuracy of the peak detection can be improved up to 99.90% and 98.59% for training and testing, respectively, as compared to the framework without feature selection adaptation. Additionally, the proposed framework based on RA-PSO offers a better and reliable classification rate as compared to standard PSO as it produces low variance model.

## 1. Introduction

The peak detection algorithms have significantly been used on different types of biological signals such as electrooculogram (EOG), electrocardiogram (ECG), and electroencephalogram (EEG). EOG signal is generated by human eye. ECG signal is generated by heart. EEG signal is generated by brain. The peak detection in the EOG signal has been used for detecting the eye blink [1, 2]. In the EOG based signal, a number of electrodes are placed around the eyes. If the eyes move in vertical direction, positive or negative peak points will arise. For the ECG signal, peak detection is typically used to detect the combination of Q, R, and S waves or the so-called QRS complex [3]. The QRS complex is a peak model for ECG signal including Q-valley point, R-peak point, and S-valley point. Other important peak points in ECG signal are P-peak point and T-peak point. The detection of the QRS complex is critical part in numerous ECG signal processing system. The different pattern of QRS complex will determine the patient heart syndrome. Additionally, the peak detection for the EEG signal has been widely used to detect P300 response [4, 5] and epilepsy response [6]. P300 is a brain response measured by electrodes covering the parietal lobe in the presence of visual and auditory stimuli. A brain with chronic disorder will respond with epilepsy. Therefore, the utilization of peak detection algorithm for the biological signals is compatible in this study.

To date, variety approaches of peak detection algorithms have been proposed. These algorithms can be categorized into four main approaches based on time domain [7–15], frequency domain [16], time-frequency domain [10, 17], and nonlinear [18]. In time domain approach, the peaks are analyzed in time. In frequency domain approach, the peaks are analyzed in frequency. In time-frequency domain approach, the peaks are analyzed in both time and frequency domain. In nonlinear approach, some statistical parameters of the peaks are analyzed. The general framework of peak detection algorithm usually involves several processes which are signal preprocessing, peak candidate detection, feature extraction, and classification. Various signal preprocessing methods have been employed such as data compression [19], wavelet transform [6], Kalman filter [20], and Hilbert transform [15]. Two methods for peak candidate detection have been used which are three point sliding window method [8] and k-point nonlinear energy operator (k-NEO) method [21]. Various feature extraction techniques have been proposed which are model-based [21], wavelet analysis [22], template matching [23], and power spectra analysis [24]. Several classifiers have been used, which are rule-based [8, 24], artificial neural network (ANN) [10, 11, 25, 26], support vector machine (SVM) [7, 27], and expert system [10]. The highlighted purposes in designing the framework are to achieve the highest performance and to reduce the computational time. Almost all studies in the EEG peak detection literature focus on the problem of detecting peaks in epileptic EEG signals. A review of peak detection algorithms that is employed to the epileptic EEG signal is presented in [28]. The peak detection is just a first step in epileptic event detection. The main goal is to determine the epileptic spikes not the whole peaks. Therefore, for an epileptic event detection system, the epileptic spike detection performance not the peak detection performance is the performance of interest.

In time domain approach, fourteen different peak features are recognized from different peak models [7–10]. The peak model is a set of peak features that represents a peak by its amplitude, width, and slope. Most algorithms [7–13, 21] in time domain approach consider different peak models and the different styles of framework. The peak model is chosen based on the experiences of EEG expert. To date, there is no any peak detection framework that automatically finds the finest existing peak model. The use of the finest peak model will give a chance for the algorithm to achieve a good performance. On the other hand, the chosen peak model is not necessarily suitable for different types of biological signal. Moreover, the finest peak model represents some meaningful information on the signal to be evaluated. Therefore, the adaptation of feature selection technique is important in this study to automatically find the finest peak model. The utilization of feature selection on peak detection algorithm will also reduce the computational time.

In this study, feature selection and classifier parameters estimation method based on standard particle swarm optimization (PSO) and random asynchronous PSO (RA-PSO) algorithm are employed. The process to find the finest peak model and classifier parameter estimation is executed simultaneously. The peak features will be evaluated by a rule-based classifier. The role of the classifier is to distinguish between peak point and non-peak point. Rule-based classifier is employed due to the ability to provide an outstanding interpretation for the obtained decisions [24]. In addition, the parameter values are tricky to be estimated manually. A PSO algorithm is considered to be appropriate for addressing the problem based on the reason in which the feature selection is a binary search problem and determination of classifier parameter is a continuous search problem [29].

### 1.1. Peak Model in Time Domain Analysis

Peak model is a set of peak features that represents a peak by its amplitude, width, and slope. In time domain analysis, fourteen different peak features are recognized from different peak models [8–10]. The earliest peak model was introduced by Dumpala et al. in 1982 [8]. The peak model comprises four features, which are (1) the amplitude between the magnitude of peak point and the magnitude of valley point at the first half wave, (2) the width between valley point of first half point and valley point at second half wave, (3) and (4) two slopes between a peak point and valley point in the first half wave and second half wave. A similar definition of the peak amplitude and slopes are also been used in [7, 11, 13].

An additional feature of peak amplitude and two features of peak width have been introduced by Acir et al. [7, 11]. The additional peak amplitude is the amplitude between the magnitude of peak point and the magnitude of valley point of the second half wave. The peak widths are the width between peak point and valley point of first half wave and second half wave. The total features that are introduced by Acir et al. are six features. Acir et al. did not use the width feature that was introduced by Dumpala et al. A similar definition of the peak amplitudes, widths, and slopes has also been used in [21]. In [21], an additional peak feature is added with a set of features that is introduced in [7, 11], which is the area of peak. However, the definition of area integration is not presented in the paper.

In addition, Liu et al. [10] have introduced eleven peak features. The proposed peak model consists of four amplitudes: (1) the amplitude between the magnitude of peak point and the magnitude of valley point at the first half wave; (2) the amplitude between the magnitude of peak point and the magnitude of valley point of the second half wave; (3) the amplitude between the magnitude of peak and the magnitude of turning point at the first half wave, and (4) the amplitude between the magnitude of peak and the magnitude of turning point at the second half wave. The turning point is defined as the point where the slope decreases more than 50% as compared to the slope of the preceding point. The model also consists of three widths: (1) the width between valley point at first half point and valley point at second half wave, (2) the width between turning point at first half wave and turning point at second half wave, and (3) the width between half point at first half wave and half point at second half wave. There are four slopes that are also measured: (1) and (2) two slopes between a peak point and valley point in the first half wave and second half wave, (3) and (4) two slopes between peak point and turning point at first half wave and second half wave.

Another peak model consists of four features, which has been proposed by Dingle et al. [9]. The peak amplitude is the difference between the peak point and the floating mean. The floating mean is the average EEG which is centered at the peak point that is also called moving average curve (MAC) [12]. The width is calculated based on the difference between the valley point at the first half wave and the valley point at the second half wave. The two slopes are the slopes between a peak point and valley point in the first half wave and second half wave. Summary of different peak models on different style of framework is briefly described in Table 1. The strength and weakness are also highlighted in Table 1. Generally, the authors claimed that the selected peak feature offers good classification performance on the proposed framework. However, the previous works did not provide the justification on the selected features.

## 2. Methodology

**Figure 1:**
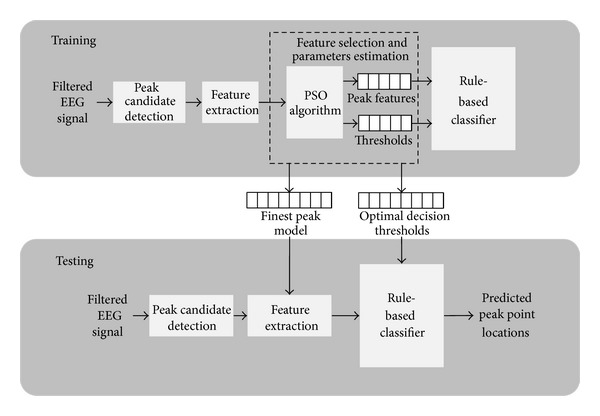
Feature selection and parameters estimation framework for peak detection algorithm.

Figure 1 shows the framework of the proposed techniques for EEG signal peak detection. There are two phases of the process which are training and testing phases. The training phase is firstly run to find the finest peak model and the optimal decision threshold values. Next, the testing phase is utilized for unseen EEG signal.

The framework can be divided into four stages: peak candidate detection, features extraction of peak candidate, feature selection and parameters estimation, and classification. In the first stage, the detection of peak candidates is performed to differentiate between a peak candidate and a non-peak candidate. The second stage is the extraction of peak candidate features. In the third stage, PSO algorithm is adapted during the training phase for feature selection and classifier parameters' estimation. Finally, the peak candidates are classified between predicted peak and predicted non-peak at particular locations by rule-based classifier.

### 2.1. Peak Candidate Detection

**Figure 2:**
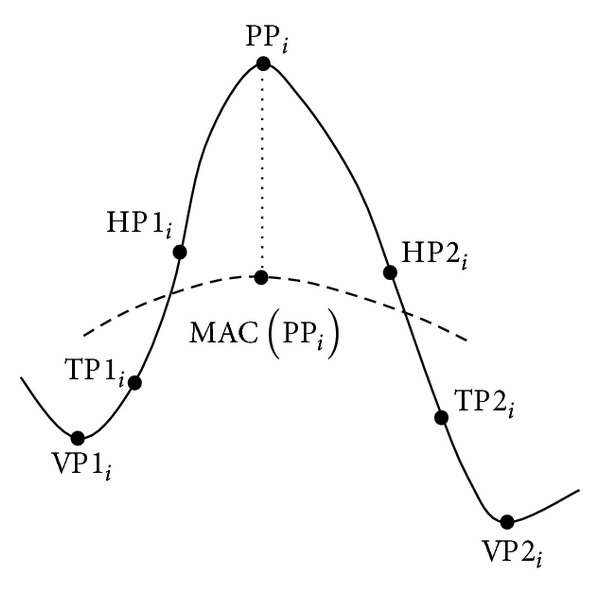
Model-based parameters.

The first step to detect peaks is to find candidate peaks. Consider a discrete-time signal, *x*(*I*), of *L* points. The *i*th candidate peak point, PP_*i*_, as shown in Figure 2, is identified using three-points sliding window method [8]. Those three-points are denoted as *x*(*I* − 1), *x*(*I*), and *x*(*I* + 1) for *I* = 1,2,…, *L*. A candidate peak point is identified when *x*(PP_*i*_ − 1) < *x*(PP_*i*_) > *x*(PP_*i*_ + 1) and two associated valley points, VP1_*i*_ and VP2_*i*_, are in between as shown in Figure 2. Both valley points exist when *x*(VP1_*i*_ − 1) > *x*(VP1_*i*_) < *x*(VP1_*i*_ + 1) and *x*(VP2_*i*_ − 1) > *x*(VP2_*i*_) < *x*(VP2_*i*_ + 1).

### 2.2. Feature Extraction

Based on the existing peak models, the total peak features are fourteen. The peak features of a peak candidate are calculated based on the eight model-based parameters as shown in Figure 2. The parameters consist of the *i*th candidate peak point, PP_*i*_, the two associated valley points, VP1_*i*_ and VP2_*i*_, the half point at first half wave (HP1_*i*_), the half point at second half wave (HP2_*i*_), the turning point at first half wave (TP1_*i*_), the turning point at second half wave (TP2_*i*_), and the moving average curve (MAC(PP_*i*_)). The peak features can be categorized into three groups; amplitude, width, and slope. There are five different amplitudes, five different widths, and four different slopes that can be calculated based on the model-based parameters. All equations and description of peak features are tabulated in Table 2. Referring to Table 3, the peak model, which is introduced by Dumpala et al. [8] and Dingle et al. [9], consists of four features. The peak model, which is specified by Acir et al. [7, 11], consists of six features. The peak model, which is specified by Liu et al. [10], consists of eleven features.

### 2.3. Feature Selection and Parameters Estimation Using Particle Swarm Optimization

In this stage, the peak features and classifier parameters are simultaneously found using two different PSO algorithms which are standard PSO and RA-PSO algorithms. At the end of this stage, the finest peak model and the optimal classifier parameters are obtained. The optimal classifier parameters represent the optimal decision threshold values.

The PSO algorithm was firstly introduced by Kennedy and Eberhart in 1995 [30]. The PSO algorithm has been numerously enhanced fundamentally [31, 32] and applied in many fields [33–35]. Fundamentally, the PSO algorithm follows several steps as described in Algorithm 1: (1) initialization, (2) calculation of the fitness function, (3) updating the personal best (*pbest*) for each particle and global best (*gbest*), (4) updating the particle's velocity and the particle's position, and (5) performing termination based on a stopping criterion.

In PSO, particles search for the best solution and update the position information from iteration to iteration. Each particle in the population consists of a vector position and vector velocity in *d* dimension. The position of particle *i* at iteration *k* is denoted as *s*_*i*_^*k*^ = {*x*_*i*,1_^*k*^, *x*_*i*,2_^*k*^, *x*_*i*,3_^*k*^,…, *x*_*i*,*d*_^*k*^}, while the velocity of particle *i* at iteration *k* is denoted as *v*_*i*_^*k*^ = {*v*_*i*,1_^*k*^, *v*_*i*,2_^*k*^, *v*_*i*,3_^*k*^,…, *v*_*i*,*d*_^*k*^}. The* pbest* of particle *i* is represented as *p*_*i*_^*k*^ = {*p*_*i*,1_^*k*^, *p*_*i*,2_^*k*^, *p*_*i*,3_^*k*^,…, *p*_*i*,*d*_^*k*^} and the* gbest* is denoted as *p*_*g*_^*k*^ = {*p*_*g*,1_^*k*^, *p*_*g*,2_^*k*^, *p*_*g*,3_^*k*^,…, *p*_*g*,*d*_^*k*^}. To obtain the updated position of a particle, *s*_*i*_^*k*+1^, each particle changes its velocity as the follows:

(1) $$\begin{array}{c} v_{i}^{k+1}=\omega v_{i}^{k}+c_{1}r_{1}\left(p_{i}^{k}-x_{i}^{k}\right)+c_{2}r_{2}\left(p_{g}^{k}-x_{i}^{k}\right), \end{array}$$

where *c*_1_ is a cognitive coefficient, *c*_2_ is a social coefficient, *r*_1_ and *r*_2_ are random values [0,1], and *ω* is a decrease inertial weight [36, 37] calculated as follows:

(2) $$\begin{array}{c} \omega =\omega_{\max}-\left(\frac{\omega_{\max}-\omega_{\min}}{k_{\max}}\right)\times k, \end{array}$$

where *ω*_max⁡_ and *ω*_min⁡_ denote the maximum and minimum values of inertia weight, respectively, and *k*_max⁡_ is the maximum iteration. Then, the particle's position is updated based on (3). Note that this equation is only valid for continuous version of PSO algorithm:

(3) $$\begin{array}{c} s_{i}^{k+1}=s_{i}^{k}+v_{i}^{k+1}. \end{array}$$

For a binary version of PSO [38], the particle position is updated based on the following equation:

(4) $$\begin{array}{c} T\left(v_{i}^{k+1}\right)=\left|\tanh\left(v_{i}^{k+1}\right)\right|, \end{array}$$

(5) $$\begin{array}{c} s_{i}^{k+1}=\{\begin{array}{cc} {\left(s_{i}^{k}\right)}^{-1} & \text{if}\;\text{rand}<T\left(v_{i}^{k+1}\right)\\ s_{i}^{k} & \text{rand}\geq T\left(v_{i}^{k+1}\right). \end{array} \end{array}$$

Equation (4) is a transfer function which is the main part of the binary version. Several studies have proven that this transfer function significantly improves the performance of the standard binary PSO. Equation (5) is used to update the particle position according to the given rules, where *s*_*i*_^*k*^ and *v*_*i*_^*k*^ represent the vector position and velocity of *i*th particle at iteration *k* and (*s*_*i*_^*k*^)^−1^ is the complement of *s*_*i*_^*k*^. The particle position maintains the current position when the velocity is lower than random value and its complement the position when the velocity is greater than random value. This method has been introduced by Mirjalili and Lewis (2013) that is also named as v-shaped transfer function [39].

Synchronous update in standard PSO algorithm indicates that all particles move to their new position after all particles are evaluated, as described in Algorithm 1. However, in RA-PSO [40], a particle immediately updates its position after it is evaluated without the need to wait until the evaluation of all particles is completed. Moreover, an *i*th particle in a population is randomly chosen with a total *N* times before *i*th particle is evaluated. *N* is the total number of particles. Some particles might be chosen more than once while some particles might not be chosen at all. The RA-PSO algorithm is described in Algorithm 2.

To perform the feature selection and parameters estimation simultaneously, both versions of PSO algorithm are employed to the standard PSO and RA-PSO algorithms.  Table 4 illustrates the representation of particle position. The *i*th particle at iteration *k*, *s*_*i*_^*k*^, in PSO represents two types of dimensions which are binary and continuous type of dimension [29], *s*_*i*_^*k*^ = {*x*_*i*,1_^*k*^, *x*_*i*,2_^*k*^,…,*x*_*i*,*d*_^*k*^, *x*_*i*,1_^*k*^, *x*_*i*,2_^*k*^,…,*x*_*i*,*D*_^*k*^}. The *d* = 1,2, 3,…, *nf* is a *d*th dimension of binary type, and the *D* = *nf* + 1, *nf* + 2, *nf* + 3,…, *nf* × 2 is a *D*th dimension of continuous type. *nf* is the total number of peak features. The particle dimension is a two times number of features. The number of thresholds is equal of the number of features.

In the initialization stage of PSO algorithm, some of the parameters are initialized: (1) the initial PSO parameters and (2) the initial particle position. The initial PSO parameters consist of the maximum inertia weight, *ω*_max⁡_, the minimum inertia weight, *ω*_min⁡_, the velocity clamping, *v*_max⁡_ the velocity vector for each particle, the* pbest* score for each particle,* gbest* score, the cognitive component, *c*_1_, and the social component, *c*_2_. The random values, *r*_1_ and *r*_2_, are randomly distributed values from 0 to 1. All particles are randomly placed within the search space.

For the calculation of fitness function, geometric mean (*Gmean*) is employed. The* Gmean* is calculated as follows:

(6) $$\begin{array}{c} \text{TPR}=\frac{\text{TP}}{\text{TP}+\text{FN}},\\ \text{TNR}=\frac{\text{TN}}{\text{TN}+\text{FP}},\\ Gmean=\sqrt{\text{TPR}\times \text{TNR}}, \end{array}$$

where true peak (TP) is a correctly detected peak point, true non-peak (TN) is a correctly detected non-peak point, false peak (FP) is a wrongly detected the non-peak point, false non-peak (FN) is a wrongly detected peak point, TPR is a true peak rate, and TNR is a true non-peak rate.

### 2.4. Rule-Based Classifier

A rule-based classifier is employed to distinguish whether the candidate peak is a true peak or true non-peak from the extracted features. Each feature has a corresponding threshold value in the classification process. Given a set of features, a true peak only can be identified if all the feature values are greater than or equal to the decision threshold values. Otherwise, the candidate peak belongs to true non-peak. The form of the rule is

(7)  IF  f1≥ th1  AND  f2≥ th2  AND…AND fM≥ thN  THEN  Candidate  Peak  is  a  True  Peak,

where *f*_*i*_ is denoted as a one of sixteen peak features, th_*i*_ is denoted as one of the decision threshold values of this peak feature, and true peak is predicted peak at a particular peak point location.

## 3. Experimental Setup

In this section, two experiments are conducted for peak detection of EEG signal. For first experiment, the framework is executed without feature selection. For second experiment, the experiment is executed with feature selection. The experimental protocols are discussed in the next subsection. The training and testing EEG signal are prepared to evaluate the performance of the proposed framework. Then, the results are discussed and analyzed.

Each experiment is conducted in 10 independent runs. For each run, 30 particles are used to perform feature selection and parameters estimation. For each particle, the total number of dimensions is depending on the number of features in a feature set. The maximum iteration was set to 1000. For the initial value of PSO parameters, the maximum inertia weight, *ω*_max⁡_, is 0.9 and the minimum inertia weight, *ω*_min⁡_, is 0.4. The cognitive component, *c*_1_, and the social component, *c*_2_, are set to 2. These values are proposed by Shi and Eberhart in 1999 [41]. The random values, *r*_1_ and *r*_2_, are randomly distributed values from 0 to 1. The velocity clamping, *v*_max⁡_, for binary version is set to 6 [39]. The velocity vector for each particle, the* pbest* score for each particle, and* gbest* score is set to 0. The parameters setting of standard PSO and RA-PSO algorithms are tabulated in Table 5.

### 3.1. Experimental Protocols

This study uses the eye movement EEG signal as a case study to evaluate the proposed framework. The observation of the eye movement EEG signal indicates that the most observable signal pattern is the peak point which signifies the brain response on eye movements. The known peak point locations through the response of the brain can be translated into an output, for example, wheelchair movement.

The experimental protocol to acquire this EEG signal was reviewed and approved by the Medical Ethic Committee (MEC) in the University of Malaya Medical Centre (UMMC). The subject gave a written consent prior to the data collection session. This EEG signal was acquired in the Applied Control and Robotic (ACR) Laboratory, Department of Electrical Engineering, Faculty of Engineering, University of Malaya, Malaysia. A healthy subject was involved voluntarily in this data collection session who is a postgraduate student in the Faculty of Engineering.

The EEG signal recording was conducted using the g.MOBIlab portable signal acquisition system. The EEG signal was recorded from C4 channel. The EEG signal of channel CZ was used as a reference. The ground electrode was located on the forehead. The electrode was placed using the 10–20 international electrode placement system. The sampling frequency was set to 256 Hz.

Before the session begins, the subject was advised to get good rest. Thus, he can give full focus during the session. The subject was also advised to wash his hair. During the data collection session, the subject was required to be ready within 0 to 4 seconds for waiting for an external cue. The cue is a command for a subject to move their eyes to the right position. Within the standby time, the subject is required not to move their eyes into a frontal position.

**Figure 3:**
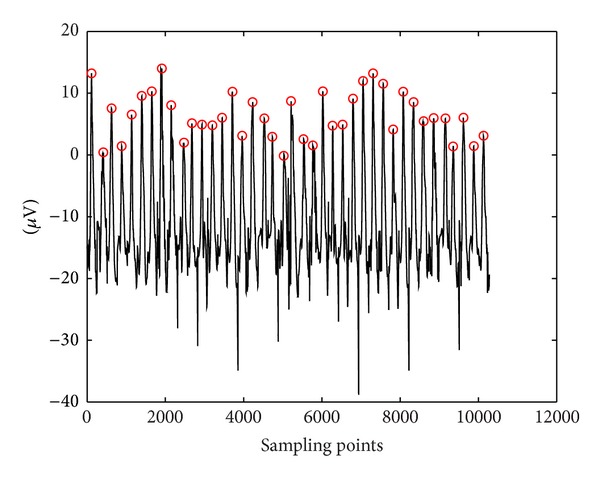
Filtered EEG signal.

When the time is exactly 5 seconds, the external cue appears on the screen monitor. The instruction allows the subject to move back their eyes in a frontal position. The external cue appears for 40 times. The total length of EEG recording is 40 seconds. As a cleanliness procedure, the electrodes and head-cap that are used in the session were washed. The filtered EEG signal is shown in Figure 3. Forty locations of definite peak points are highlighted in the red circle. The next process is to prepare the training and testing data.

From the data collection, 40 definite peak point locations have been identified by EEG expert. In 40-second signal there are 10240 sampling points, *x*(*I*). There are only 40 peak points and the remaining of 10200 sampling points are the non-peak points. For preparing the training and testing signal, the training signal is selected from 1 to 5120 sampling points while the remaining EEG signal is used for testing signal. The signal specification is summarized in Table 6.

## 4. Results and Discussions

To evaluate the proposed framework for training and testing phase, four different measures are used including the average* Gmean*, the maximum* Gmean*, the minimum* Gmean*, and the standard deviation (STDEV).

### 4.1. Results of Peak Detection Algorithm without Feature Selection

Four peak models are employed for evaluating the peak models performance in the proposed framework. The training and testing performance based on those four different measures for each model is shown in Table 7. The standard PSO algorithm is used to find the optimal threshold values for each peak model. The obtained results for each peak model are compared with the results of peak detection algorithm and the feature selection framework based on standard PSO. Notably, in this section, only standard PSO is considered in the peak detection algorithm without feature selection framework.

Referring to Table 7, the training performance for average, maximum, minimum, and STDEV is 84.01%, 89.15%, 80.58%, and 4.43% for Dumpala et al.'s peak model; 74.4%, 80.59%, 67.08%, and 3.71% for Acir et al.'s peak model; and 90.98%, 94.76%, 83.66%, and 5.51% for Dingle et al.'s peak model, respectively. The testing performance for average, maximum, minimum, and STDEV is 81.22%, 91.83%, 74.15%, and 9.13% for Dumpala et al.'s peak model; 68.59%, 77.43%, 54.77%, and 6.97% for Acir et al.'s peak model; and 88.78%, 94.75%, 77.44%, and 7.98% for Dingle et al.'s peak model, respectively.

Overall, the average performance of the training phase for Dumpala et al.'s peak model, Acir et al.'s peak model, and Dingle et al.'s peak model is greater than the average performance of their testing phase. However, for the peak model, Liu et al.'s peak model, will give zero percent performance for training and testing phase. This result indicates the limitation of rule-based classifier when dealing with both feature sets. During the training process on the feature sets, the particles in the PSO algorithm do not meet the optimum decision threshold values and the particles might also be trapped at local optima. Based on the preceding rule, a true peak only can be identified if all the feature values are greater than or equal to the decision threshold values. So, if one of the feature values does not satisfy the decision threshold value, the classifier will decide the peak candidate as a non-peak point. When this happens to all peak candidates, the TP is equal to zero.* Gmean* will give zero percent performance even if TN is equal to some values. The end results indicate the employment of the presented rule is only valid for Dumpala et al.'s peak model, Acir et al.'s peak model, and Dingle et al.'s peak model.

Compared to the test average performance of the peak models, the highest test performance is obtained by Dingle et al.'s peak model, which is 88.78%, then follows by Dumpala et al.'s peak model, which is 81.22%. The worst test performance is obtained by Acir et al.'s peak model, which is 68.59%. It can be concluded: from the findings of experimental results, the finest peak model for the filtered EEG signal is Dingle et al.'s peak model, and the worst peak model for the filtered EEG signal is Acir et al.'s peak model. True peak rate and true non-peak rate of test performance are shown in Table 8. It can be concluded that, from the finding experimental results, the chosen peak models limit the designed framework to obtain the best accuracy. Therefore, the feature selection technique using standard PSO is employed into the designed framework.

### 4.2. Results of Peak Detection Algorithm with Feature Selection

The results of peak detection algorithm with feature selection are categorized into two subsections which are the results of feature selection using standard PSO and the results of feature selection using RA-PSO. Also, the results from the two PSO algorithms in the proposed framework are discussed.

#### 4.2.1. Feature Selection Using Standard PSO

The feature sets of 10 runs using the standard PSO algorithm are shown in Table 9. The result shows the variety of the optimal combination of features that give the higher classification performance, mostly higher than 99.69%. The maximum training accuracy is 99.98%. The most significant peak feature is the feature *f*_5_ because all the 10 runs appear as a selected feature by PSO. Feature *f*_5_ is the amplitude that is calculated from the difference between peak points (PP) and moving average curve (MAC). Another most significant feature is feature *f*_2_, which is the calculated amplitude between a peak point and valley point at the second haft wave. The feature *f*_6_ is chosen 4 times. The feature *f*_6_ is chosen 4 times. The features *f*_4_ and *f*_9_ are chosen 2 times. The feature *f*_10_ is only selected at 9th run.

Based on the results in Table 9, the combination of peak features (*f*_2_, *f*_5_, and *f*_6_) appears 4 times, the combination of peak features (*f*_2_, *f*_5_, and *f*_9_) appears 2 times, and the combination of peak features (*f*_2_ and *f*_5_) appears 2 times. Therefore, there are 3 optimal combinations of features that can be chosen.

Table 10 has the optimal threshold values for the optimal combination of the features. The threshold values are selected based on the selected peak features that are highlighted in the table.

The average of training and testing results of 10 runs using standard PSO algorithm is tabulated in Table 11. The results of standard PSO show the average training accuracy is 99.91%. The maximum training accuracy is 99.98%. The minimum training accuracy is 99.69%, and the standard deviation is 8.07%. On the other hand, the testing accuracy is 93.73%. The maximum testing accuracy is 99.92%. The minimum testing accuracy is 77.41%.

In terms of peak and the non-peak rate (TP and TN) for training results, the classifier accurately predicted all 20 peak points and 5113 non-peak points. The results also show that the classifier misclassified 27 non-peak points. The maximum of the true peak point is 20 and true non-peak point is 5118. The minimum of true peak point is 20, and true non-peak point is 5109.

For testing results, the classifier accurately predicted 18 peak points and 5110 non-peak points. The maximum of the true peak point is 20 and true non-peak point is 5114. The minimum of true peak point is 12 and true non-peak point is 5106. In general, the average testing result that corresponds to the selected peak features using the proposed feature selection framework is greater than the average testing result of Dingle's peak model which is 93.73% and 88.78%. The feature set of the Dingle's peak model is *f*_5_, *f*_6_, *f*_11_, and *f*_12_ while the feature set that gives a higher training performance in this experiment is *f*_2_ and *f*_5_.

However, the proposed framework based on standard PSO produces slightly high variance model as it measures from the STDEV index. The STDEV is evaluated for measuring the algorithm consistency where lowest STDEV value indicates a good generalization algorithm. Based on the results of the STDEV in Table 13, the STDEV values of the standard PSO are 8.07% and 7.18% for training and testing, respectively. This results show that the high standard deviation of the accuracy is recorded between maximum and minimum of classification rate. The experimental results are reasonable due to the limitation of the standard PSO algorithm.

#### 4.2.2. Feature Selection Using RA-PSO

Table 12 shows the feature selection results of 10 runs based on the RA-PSO algorithm. The feature set was highlighted of each run. The threshold values for all selected features are also given in Table 13. The highest* Gmean* value of training phase is 99.91%. The significant peak features are *f*_5_ and *f*_8_. The corresponding threshold values are 9.20 and 4. Note that feature *f*_5_ is the amplitude that is calculated from the difference between peak points (PP) and moving average curve (MAC). Another most significant feature is feature *f*_8_, which is the width between peak point and valley point of second half wave. The features *f*_2_, *f*_4_, and *f*_8_ are chosen 3 times. The feature *f*_12_ is only selected at second run.

Three similar results were obtained out of ten runs. Other significant feature sets that are obtained in this result are the combination of peak features (*f*_2_ and *f*_5_) and (*f*_4_ and *f*_5_). These feature sets also appear 3 times.

Table 14 shows the average training and testing results of 10 runs with feature selection using RA-PSO algorithm. The average* Gmean* value of the RA-PSO algorithm is 99.90% and 98.59% for training and testing, respectively. The maximum* Gmean* value of the RA-PSO algorithm is 99.91% and 99.86% for training and testing, respectively. The minimum* Gmean* value of the RA-PSO algorithm is 99.87% and 97.33% for training and testing, respectively.

In terms of peak and the non-peak rate (TP and TN) for training results, the classifier accurately predicted all 20 peak points and 5110 non-peak points. The results also show that the classifier misclassified 30 non-peak points. The maximum of the true peak point is 20 and true non-peak point is 5111. The minimum of true peak point is 20 and true non-peak point is 5107.

For testing results, the classifier accurately predicted 19 peak points and 5106 non-peak points. The maximum of the true peak point is 20 and true non-peak point is 5107. The minimum of true peak point is 19 and true non-peak point is 5103.

As compared to the framework, using standard PSO, RA-PSO is found to offer lower variance model. The recorded STDEV values of the RA-PSO are 1.15% and 1.33% for training and testing, respectively. Therefore, the RA-PSO may offer a reliable and reasonable model as compared to standard PSO with consistent classification rate.

## 5. Conclusions

In this study, the framework of feature selection and parameters estimation is proposed for EEG signal peak detection algorithm. The proposed framework involves peak candidate detection, feature extraction, feature selection, and classification. The framework is developed based on PSO algorithm and a rule-based classifier. In general, the binary PSO based algorithm was utilized for selecting the peak features while the continuous PSO based algorithm was utilized for optimizing the classifier parameters. Two PSO based algorithms are employed in the proposed framework: (1) standard PSO and (2) RA-PSO. Fourteen peak features were employed in this study. All these peak features were taken from the existing peak models in the time domain approach. The available peak features are then automatically selected in combinatorial form using the proposed framework. Based on the experiment results of peak detection algorithm without feature selection, the best peak model is Dingle et al.'s [9] peak model where the highest performance obtained is 88.78%. Meanwhile, the experimental results with feature selection show the proposed framework with standard PSO can further improve the Dingle et al.'s model. However, the recorded results are inconsistent due to high variances of the classification accuracy. The unreliability of the standard PSO can be further improved based on the proposed framework using RA-PSO. In general, the proposed feature selection technique offers a better performance as compared to any peak models without feature selection. For future work, the proposed framework will be employed in more case studies and will invent more classification methods.

## Figures and Tables

**Algorithm 1:**
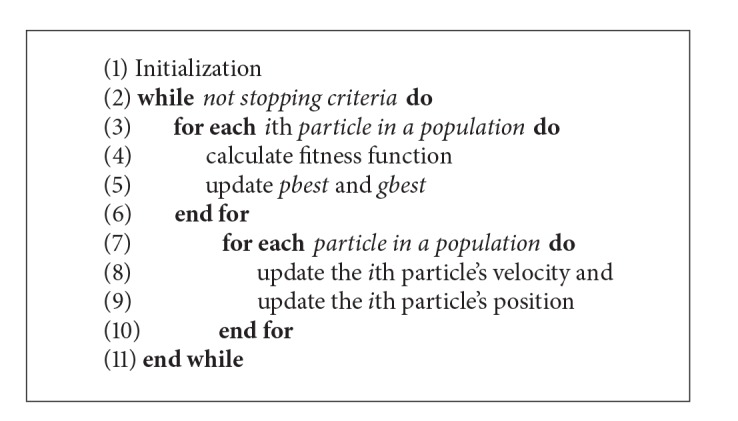
Standard PSO Algorithm.

**Algorithm 2:**
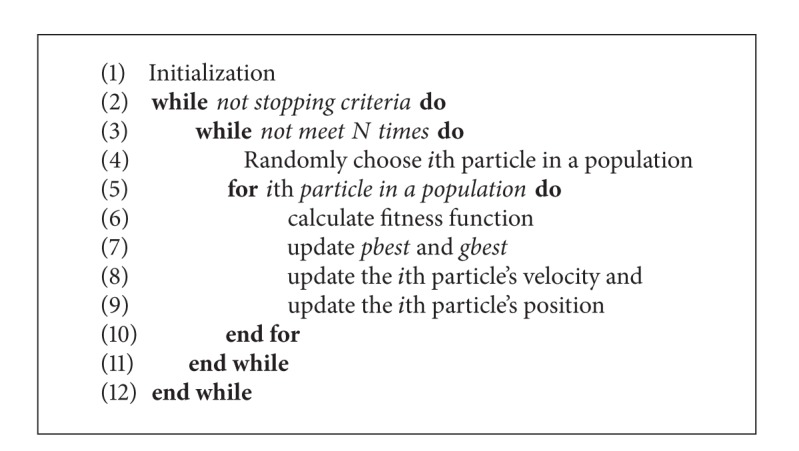
Random Asynchronous PSO (RA-PSO).

**Table 1:** Summary of different peak models on different style of framework.

Peak model	Type of signal	Description of framework
Dumpala et al. (1982) [8]	Electrical control activity (ECA)	The theory of maxima and minima using three-point sliding window approach has been applied to detect a candidate peak. Two flowcharts of peak detection have been proposed. A predicted peak can be identified if the feature values satisfied the decision threshold values. The strength and weakness of the proposed approach are described as follows: (1) strength: the authors claimed that the proposed peak detection algorithm can be used for other biological signals, (2) weakness: the utilization of peak-to-peak amplitude on the peak model is hard to distinguish between noise and actual peak. In addition, large variation of peak width in the signal may drop the classification performance.

Dingle et al. (1993) [9]	Epileptic EEG	Based on the defined peak model, the features are grouped into two: (1) epileptiform transient parameters and (2) background activity parameters. Two-threshold systems have been employed to detect a candidate peak or candidate epileptiform transient. Expert system which considered both spatial and temporal contextual information has been used to reject the artifacts and classify the transient events. The strength and weakness of the proposed approach are described as follows: (1) strength: moving average amplitude is good in rejecting false peak points. The employed features are claimed to offer good performance in the proposed expert system, (2) weakness: inconsistency of feature slope information as the proposed work claimed that the proposed framework fails to provide slope information.

Liu et al. (2002) [10]	Epileptic EEG	Wavelet transform has been used to decompose the EEG signal. Based on the decomposed signals and the defined peak model, seven features are calculated. These features are used as the input of ANN classifier. Expert system which considered both spatial and temporal contextual information has been used to reject the artifact. Several heuristic rules have been employed to distinguish the type of artifact. After all artifacts are recognized and rejected, the decision will be made to classify the epileptic events. The strength and weakness of the proposed approach are described as follows: (1) strength: the employed features is claimed to offer good performance in the proposed expert system, (2) weakness: it considers that almost all the features may deteriorate the classification performance.

Acir et al. (2005) [11]	Epileptic EEG	A three-stage procedure based on ANN is proposed for the detection of epileptic spikes. The EEG signal is transformed into time-derivative signal. Several rules have been used to detect a peak candidate. The features of peak candidate are calculated based on the defined peak model. These features are fed into two discrete perceptron classifiers to classify into three groups: definite peak, definite non-peak, and possible/possible non-peak. The peak that belongs in the third group is going to be further processed by nonlinear classifier. The strength and weakness of the proposed approach are described as follows: (1) strength: the employed features are claimed to offer good performance in the proposed system, (2) weakness: inconsistency of feature slope information as the proposed work claimed that the proposed framework fails to provide slope information.

Acir (2005) [26]	Epileptic EEG	A two-stage procedure based on a modified radial basis function network (RBFN) is proposed for the detection of epileptic spikes. The EEG signal is transform into time-derivative signal. Several rules have been used to detect a peak candidate. The features of peak candidate are calculated based on the defined peak model. These features are fed into discrete perceptron classifiers to classify into two groups: definite non-peak and peak-like non-peak. The peak that belongs to the second group requires further process by modified RBFN classifier. The strength and weakness of the proposed approach are described as follows: (1) strength: the employed features are claimed to offer good performance in the proposed system, (2) weakness: inconsistency of feature slope information as the proposed work claimed that the proposed framework fails to provide slope information.

Liu et al. (2013) [21]	Epileptic EEG	A two-stage procedure is proposed for the detection of epileptic spike. k-NEO has been used to detect a candidate peak. The peak features are calculated based on the defined peak model. These features are then used as the input of the AdaBoost classifier. The strength and weakness of the proposed approach are described as follows: (1) strength: the peak model considers feature based on peak area, (2) weakness: the definition of area integration is not presented in the paper.

**Table 2:** Equations and descriptions of peak features.

Peak feature	Equation	Description
Amplitudes	*f*_1_ = |*x*(PP_*i*_) − *x*(VP1_*i*_)|	Amplitude between the magnitude of peak and the magnitude of valley at the first half wave
	*f*_2_ = |*x*(PP_*i*_) − *x*(VP2_*i*_)|	Amplitude between the magnitude of peak and the magnitude of valley of the second half wave
	*f*_3_ = |*x*(PP_*i*_) − *x*(TP1_*i*_)|	Amplitude between the magnitude of peak and the magnitude of turning point at the first half wave
	*f*_4_ = |*x*(PP_*i*_) − *x*(TP2_*i*_)|	Amplitude between the magnitude of peak and the magnitude of turning point at the second half wave
	*f*_5_ = |*x*(PP_*i*_) − MAC(PP_*i*_)|	Amplitude between the magnitude of peak and the magnitude of moving average

Widths	*f*_6_ = |VP1_*i*_ − VP2_*i*_|	Width between valley point of first half point and valley point at second half wave
	*f*_7_ = |PP_*i*_ − VP1_*i*_|	Width between peak point and valley point at first half wave
	*f*_8_ = |PP_*i*_ − VP2_*i*_|	Width between peak point and valley point of second half wave
	*f*_9_ = |TP1_*i*_ − TP2_*i*_|	Width between turning point at first half wave and turning point at the second half wave
	*f*_10_ = |HP1_*i*_ − HP2_*i*_|	Width between half point of first half wave and half point of second half wave

Slopes	$f_{11}=\left|\frac{x({\text{PP}}_{i})-x({\text{VP1}}_{i})}{{\text{PP}}_{i}-{\text{VP1}}_{i}}\right|$	Slope between a peak point and valley point at the first half wave
	$f_{12}=\left|\frac{x({\text{PP}}_{i})-x({\text{VP2}}_{i})}{{\text{PP}}_{i}-{\text{VP1}}_{i}}\right|$	Slope between a peak point and valley point at the second half wave
	$f_{13}=\left|\frac{x({\text{PP}}_{i})-x({\text{TP1}}_{i})}{{\text{PP}}_{i}-{\text{TP1}}_{i}}\right|$	The slope between peak point and turning point at the first half wave
	$f_{14}=\left|\frac{x({\text{PP}}_{i})-x({\text{TP2}}_{i})}{{\text{PP}}_{i}-{\text{TP2}}_{i}}\right|$	The slope between peak point and turning point at the second half wave


**Table 3:** List of different peak models and sets of features.

Peak model	Set of features	Number of features
Dumpala et al. (1982) [8]	*f*_1_, *f*_6_, *f*_11_, *f*_12_	4
Acir et al. (2005) [7, 11, 26]	*f*_1_, *f*_2_, *f*_7_, *f*_8_, *f*_13_, *f*_14_	6
Liu et al. (2002) [10]	*f*_1_, *f*_2_, *f*_3_, *f*_4_, *f*_6_, *f*_9_, *f*_10_, *f*_11_, *f*_12_, *f*_13_, *f*_14_	11
Dingle et al. (1993) [9]	*f*_5_, *f*_6_, *f*_11_, *f*_12_	4

**Table 4:** Representation of particle position.

Particle	Peak features (binary type)				Thresholds (continuous type)			
	1	2	⋯	*nf*	*nf* + 1	*nf* + 2	⋯	*nf* × 2
*s*_*i*_^*k*^	*x*_*i*,1_^*k*^	*x*_*i*,2_^*k*^	⋯	*x*_*i*,*d*_^*k*^	*x*_*i*,1_^*k*^	*x*_*i*,2_^*k*^	⋯	*x*_*i*,*D*_^*k*^

**Table 5:** Parameters setting of standard PSO and RA-PSO algorithms.

Initial PSO parameters	
Parameters	Value
Decrease inertia weight, *ω*	0.9~0.4
Cognitive component, *c*_1_	2
Social component, *c*_2_	2
Random value, *r*_1_ and *r*_2_	Random number [0, 1]
Velocity vector for each particle	0
Initial *pbest* score for each particle	0
Initial *gbest* score	0
Range of search space for *nf* + 1 to *nf* + 5	$\begin{array}{cc} {}[0 & 30] \end{array}$
Range of search space for *nf* + 6 to *nf* + 12	$\begin{array}{cc} {}[0 & 781.25] \end{array}$
Range of search space for *nf* + 13 to *nf* × 2	$\begin{array}{cc} {}[0 & 24.16] \end{array}$

**Table 6:** Signal specifications.

Specification	Channel C4
Total sampling point	10240
Total length signal (second)	40
Number of peak points in the signal	40
Sampling frequency (Hz)	256

**Table 7:** Training and testing performance of peak detection for each peak model (without feature selection).

Peak model	Training (%)				Testing (%)			
	Average	Max	Min	STDEV	Average	Max	Min	STDEV
Dumpala et al. (1982) [8]	84.01	89.15	80.58	4.43	81.22	91.83	74.15	9.13
Acir et al. (2005) [7, 11, 26]	74.4	80.59	67.08	3.71	68.59^worst^	77.43	54.77	6.97
Liu et al. (2002) [10]	0	0	0	0	0	0	0	0
Dingle et al. (1993) [9]	**90.98**	**94.76**	**83.66**	**5.1**	**88.78** ^**best**^	**94.75**	**77.44**	**7.98**

**Table 8:** TPR and TNR test results for EEG signal (without feature selection).

Peak model	TPR (%)	TNR (%)
Dumpala et al. (1982) [8]	65.0	99.7
Acir et al. (2005) [7, 11, 26]	50.0	99.9
Liu et al. (2002) [10]	0.0	0.0
Dingle et al. (1993) [9]	**80.0**	**99.3**

**Table 9:** Training results: the feature sets of 10 runs using standard PSO.

Run	Peak features													
	Amplitudes					Widths					Slopes			*Gmean* (%)
	*f*_1_	*f*_2_	*f*_3_	*f*_4_	*f*_5_	*f*_6_	*f*_7_	*f*_8_	*f*_9_	*f*_10_	*f*_11_	*f*_12_	*f*_13_-*f*_14_	
#1	0	*1 *	0	0	*1 *	0	0	0	*1 *	0	0	0	0	99.89
#2	0	0	0	*1 *	*1 *	0	0	0	0	0	0	0	0	99.91
#3	0	*1 *	0	0	*1 *	0	0	0	*1 *	0	0	0	0	99.69
#4	0	*1 *	0	0	*1 *	*1 *	0	0	0	0	0	0	0	99.92
#5	0	*1 *	0	0	*1 *	*1 *	0	0	0	0	0	0	0	99.91
#6	0	*1 *	0	0	*1 *	0	0	0	0	0	0	0	0	99.95
#7	0	*1 *	0	0	*1 *	*1 *	0	0	0	0	0	0	0	99.94
#8	0	*1 *	0	0	*1 *	*1 *	0	0	0	0	0	0	0	99.91
#9	0	0	0	1	*1 *	0	0	0	0	*1 *	0	0	0	99.96
#10	0	*1 *	0	0	*1 *	0	0	0	0	0	0	0	0	**99.98**

**Table 10:** Training results: the optimal decision threshold values of 10 runs using standard PSO.

Run	Optimal threshold values												
	th_1_	th_2_	th_3_	th_4_	th_5_	th_6_	th_7_	th_8_	th_9_	th_10_	th_11_	th_12_	th_13_-th_14_
#1	—	*0.40 *	—	—	*9.07 *	—	—	—	*9 *	—	—	—	—
#2	—	—	—	*0.27 *	*9.20 *	—	—	—	—	—	—	—	—
#3	—	*1.24 *	—	—	*9.27 *	—	—	—	*17 *	—	—	—	—
#4	—	*0.37 *	—	—	*8.93 *	*12 *	—	—	—	—	—	—	—
#5	—	*0.43 *	—	—	*9.18 *	*12 *	—	—	—	—	—	—	—
#6	—	*1.25 *	—	—	*11.34 *	—	—	—	—	—	—	—	—
#7	—	*0.93 *	—	—	*9.07 *	*11 *	—	—	—	—	—	—	—
#8	—	*0.38 *	—	—	*9.10 *	*8 *	—	—	—	—	—	—	—
#9	—	—	—	*0.43 *	*9.13 *	—	—	—	—	*8 *	—	—	—
#10	—	*0.90 *	—	—	*10.07 *	—	—	—	—	—	—	—	—

**Table 11:** Average training and testing results of 10 runs with feature selection using standard PSO.

Algorithm	Results	Training					Testing				
		*Gmean* (%)	TN	FP	TP	FN	*Gmean* (%)	TN	FP	TP	FN
Standard PSO	AVG	*99.91 *	*5113 *	*27 *	*20 *	*0 *	*93.73 *	*5110 *	*30 *	*18 *	*2 *
	MAX	99.98	5118	22	20	0	99.92	5114	26	20	0
	MIN	99.69	5109	31	20	0	77.41	5106	34	12	8
	STDEV	8.07					7.18				

**Table 12:** Training results: the feature sets of 10 runs using RA-PSO.

RA-PSO	Peak features													
	Amplitudes					Widths					Slopes			*Gmean* (%)
Run	*f*_1_	*f*_2_	*f*_3_	*f*_4_	*f*_5_	*f*_6_	*f*_7_	*f*_8_	*f*_9_	*f*_10_	*f*_11_	*f*_12_	*f*_13_-*f*_14_	
**#1**	**0**	**0**	**0**	**0**	***1***	**0**	**0**	***1***	**0**	**0**	**0**	**0**	**0**	**99.91**
#2	0	0	0	0	*1 *	0	0	0	0	0	0	*1 *	0	99.87
#3	0	0	0	*1 *	*1 *	0	0	0	0	0	0	0	0	99.90
#4	0	0	0	*1 *	*1 *	0	0	0	0	0	0	0	0	99.90
**#5**	**0**	**0**	**0**	**0**	***1***	**0**	**0**	***1***	**0**	**0**	**0**	**0**	**0**	**99.91**
#6	0	*1 *	0	0	*1 *	0	0	0	0	0	0	0	0	99.90
#7	0	*1 *	0	0	*1 *	0	0	0	0	0	0	0	0	99.90
#8	0	0	0	*1 *	*1 *	0	0	0	0	0	0	0	0	99.90
#9	0	*1 *	0	0	*1 *	0	0	0	0	0	0	0	0	99.90
**#10**	**0**	**0**	**0**	**0**	***1***	**0**	**0**	***1***	**0**	**0**	**0**	**0**	**0**	**99.91**

	AVERAGE *Gmean *													99.90

**Table 13:** Training results: the optimal decision threshold values of 10 runs using RA-PSO.

Run	Optimal threshold values using RA-PSO												
	th_1_	th_2_	th_3_	th_4_	th_5_	th_6_	th_7_	th_8_	th_9_	th_10_	th_11_	th_12_	th_13_-th_14_
**#1**	—	—	—	—	***9.20***	—	—	***4***	—	—	—	—	—
#2	—	—	—	—	*9.21 *	—	—	—	—	—	—	*0.6 *	—
#3	—	—	—	*0.38 *	*9.21 *	—	—	—	—	—	—	—	—
#4	—	—	—	*0.22 *	*9.20 *	—	—	—	—	—	—	—	—
**#5**	—	—	—	—	***9.20***	—	—	***4***	—	—	—	—	—
#6	—	*0.36 *	—	—	*9.04 *	—	—	—	—	—	—	—	—
#7	—	*0.39 *	—	—	*9.22 *	—	—	—	—	—	—	—	—
#8	—	—	—	*0.25 *	*9.20 *	—	—	—	—	—	—	—	—
#9	—	*0.27 *	—	—	*9.19 *	—	—	—	—	—	—	—	—
**#10**	—	—	—	—	***9.20***	—	—	***4***	—	—	—	—	—

**Table 14:** Average training and testing results of 10 runs with feature selection using RA-PSO.

Algorithm	Results	Training					Testing				
		*Gmean* (%)	TN	FP	TP	FN	*Gmean* (%)	TN	FP	TP	FN
RA-PSO	AVG	***99.90***	***5110***	***30***	***20***	***0***	***98.59***	***5106***	***34***	***19***	***1***
	MAX	99.91	5111	29	20	0	99.86	5107	33	20	0
	MIN	99.87	5107	33	20	0	97.33	5103	37	19	1
	STDEV	1.15					1.33				
